# Measurement of Nonlinear Poisson’s Ratio of Thermoplastic Polyurethanes under Cyclic Softening Using 2D Digital Image Correlation

**DOI:** 10.3390/polym13091498

**Published:** 2021-05-06

**Authors:** Yi-Xian Xu, Jia-Yang Juang

**Affiliations:** Department of Mechanical Engineering, National Taiwan University, Taipei 10617, Taiwan; r08522503@ntu.edu.tw

**Keywords:** cyclic softening, thermoplastic polyurethane (TPU), 2D-DIC, Poisson’s ratio

## Abstract

Thermoplastic polyurethanes (TPUs) and other elastomers are widely used in many applications for the advantages they provide in terms of high elasticity, lightness, resistance to breakage, and impact resistance. These materials exhibit strong hysteresis in the large strain stress-strain behavior, known as cyclic softening or the Mullins effect. Despite the extensive studies on this phenomenon and the importance of Poisson’s ratio, how the Poisson’s ratio of these materials changes during cyclic uniaxial tests is still unclear. Here, we measure the nonlinear Poisson’s ratio of TPU and investigate its correlation with cyclic softening using two-dimensional digital image correlation (2D-DIC) combined with the reference sample compensation (RSC) method. This accuracy-enhanced method can effectively eliminate the measurement errors induced by the unavoidable out-of-plane displacements and lens distortion. We find that the Poisson’s ratio of TPUs also exhibits large hysteresis in the first cycle and then approaches a steady state in subsequent cycles. Specifically, it starts from a relatively low value of 0.45 ± 0.005 in the first loading, then increases to 0.48 ± 0.005 in the first unloading, and remains largely constant afterward. Such a change in the Poisson’s ratio results in a slight volume increase (≈1%) at a maximum strain of 17.5%. Our findings are useful for those who use finite element method to analyze the mechanical behavior of TPU, and shed new light on understanding the physical origin of cyclic softening.

## 1. Introduction

Soft materials are widely used in various fields, such as bioelectronics [1,2], materials science [3,4], shape morphing [5,6], and robotics [7,8,9,10,11], because of their merits of lightness, resistance to breakage, and impact resistance. In recent years, soft robotics has emerged as a viable alternative and has attracted a great deal of attention [7,8,9]. Unlike conventional rigid robots, soft robots use soft materials as the main structural materials and have benefited greatly from the advances in additive manufacturing technology using 3D printing. The use of 3D printing allows for the rapid prototyping of diverse designs, and significantly speeds up the design process. Among the various 3D printing materials, thermoplastic polyurethane (TPU), a type of elastomer, is frequently used in soft robotics as it offers the desirable elastic characteristics of rubber but can be processed as a thermoplastic [12]. However, TPU and other rubber-like materials are known to become softer after the first deformation, referred to as cyclic softening or the Mullin’s effect [12,13]. Several theories have been proposed but still no general agreement has been reached either on the physical source or the mechanical modeling of this effect [14]. Cyclic softening can affect the performance of soft robots, and should be carefully considered in design [10]. Although the stress-strain behavior of TPU and its cyclic softening have been studied for many years [12], how its Poisson’s ratio behaves during cyclic uniaxial loading is still unknown.

Poisson’s ratio is one of the most fundamental material properties of solids, and is defined as the negative quotient of the transverse strain to the axial strain in the infinitesimal uniaxial extension of a homogeneous isotropic body. The value ranges from −1.0 to + 0.5, based on thermodynamic considerations of strain energy in the theory of elasticity [1]. This definition can be extended into the nonlinear regime of large strains for elastomers [15,16]. Although Poisson’s ratio is a constant in small strains, it is a scalar function, the Poisson function, of the stretch ratio in large strains. Additionally, for a nonlinear elastic material, the Poisson’s ratio may vary with the stretch and can be expressed using different strain measures, such as Green (Lagrangian), Hencky (logarithmic or true), and Biot (nominal or engineering) strains [17]. Moreover, in practice, linear elasticity with Young’s modulus and Poisson’s ratio is often used to design and analyze structures of elastomers and rubber-like materials for its simplicity and rapid convergence in finite element analysis. It is therefore of great importance to measure the nonlinear Poisson’s ratio of TPU in large strains.

The accurate and precise measurement of the Poisson’s ratio of soft materials in large strains is challenging, since conventional methods such as strain gauge may affect the measurement due to the added weight and stiffness [18]. By contrast, digital image correlation (DIC) is a novel choice that uses an easy-to-implement but effective optical technique to achieve non-contact measurement [19,20]. DIC divides a patterned material surface into small sections and tracks their displacements from the initially undeformed image to successively analyzed images throughout the deformation. The strain field can then be calculated from the measured displacement field to a high degree of accuracy [21]. Despite the powerful function of DIC, the accuracy of the measurement may still be affected by out-of-plane translation and rotation of the specimen, as well as lens distortion. To estimate the errors and enhance the accuracy, intensive studies have been conducted. For example, Hoult et al. [22] and Zhu et al. [23] presented correction schemes by recording both front and rear surfaces of a specimen. By averaging the two strain results of the front and rear surface, the strains due to out-of-plane motion can be eliminated. Pan et al. [19,24,25] presented the reference sample compensation (RSC) method by adding a thin hollow compensation specimen (referred to as a region of compensation, ROC). The ROC is attached to the surface of the testing specimen and is undeformed during the test. By compensating for the error calculated from the ROC, all errors can be eliminated. With these methods, high-accuracy 2D-DIC measurement can be realized by using low-cost common lenses and cameras [26].

Here, we aim to measure the nonlinear Poisson’s ratio of TPU in large strains during a quasi-static cyclic tensile test. Toward this end, several discussions are presented. First, the RSC method is applied to 2D-DIC, and its improvement is quantified. Second, the nonlinear Poisson’s ratios represented by Biot strain and Hencky strain are compared. Third, we measure the hysteresis of nonlinear Poisson’s ratio during the first cycle and estimate the TPU’s volume change.

## 2. Materials and Methods

### 2.1. Specimens

**Figure 1:**
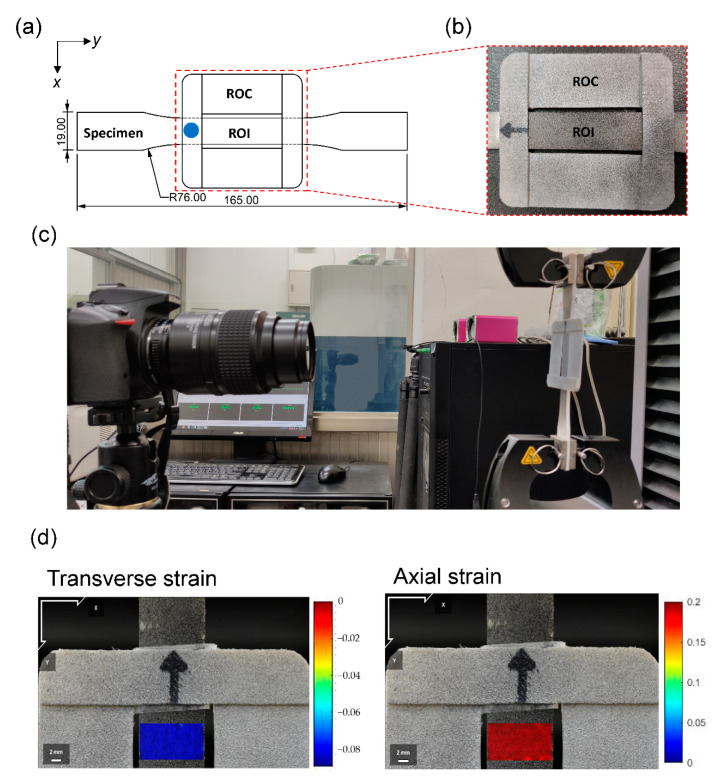
(**a**) Dimensions of the specimen (ASTM D638), the ROI, and the ROC. The blue dot indicates the attachment location. (**b**) A close-up image near the ROC and the ROI. (**c**) Experimental setup of the cyclic test. (**d**) Representative Green strain fields measured by DIC at a stretch ratio of 1.186. ROI: region of interest; ROC: region of compensation.

The investigated material was thermoplastic polyurethane (TPU, NinjaTek, Ninjaflex 85A, PA, USA), which can be 3D-printed and is widely used in soft robotics. We used a 3D printer (FlashForge, Creator Pro 2016, Zhejiang, China) to directly print the elastic specimen, avoiding the need for complex molding or assembly. For all printing in this study, the temperatures of the liquefier and build plate were respectively adjusted to 235 °C and 50 °C, and the printing speed was 30 mm/s. The nozzle was 0.25 mm and each printing layer was 0.15 mm thick. There were three perimeter shells in each layer. To achieve a filling density of 100%, outline overlap was set to 25% and 10 layers in both the top and bottom layers were applied. The infill angle was fixed at 45° and −45°. The test specimens (165 × 13 × 3 mm^3^) for cyclic tensile tests were fabricated according to ASTM D638 [27] as shown in Figure 1a.

### 2.2. Experimental Setup

The cyclic tensile tests were performed at room temperature, using a tensile machine (Criterion 42.503 Test System, MTS, MN, USA) in the displacement-controlled mode. To observe the cyclic softening in a quasi-static state, strains were applied at a cross-head speed of 5 mm/min and 10 mm/min, according to ASTM D638, to approximately 20% strain. A 0.1-mm pre-stretch was applied to eliminate the bending stress or pre-stress in the specimens. A five-cycle test was selected because the stress-strain curve was known to reach an equilibrium after approximately four cycles [12]. The total test time was roughly 40 min for each specimen at the rate of 5 mm/min.

The load data was obtained from the machine readings and the strain field was measured on the surface of each specimen by a DIC system. An open-source 2D Digital Image Correlation (Ncorr v1.2, Georgia Institute of Technology, GA, USA) analysis software program was used [28]. To measure the strain with the DIC, a random pattern of small black and white speckles was sprayed on the specimens, allowing the DIC to track relative displacements. We used a camera (D5500, Nikon, Japan) with a microlens (AF-S MICRO NIKKOR 105 mm 1:2.8 G ED, Nikon, Japan) to obtain images of the test section at the center of the specimen, that is, the region of interest, ROI (Figure 1). Video footage was taken during the entire test at 25 fps at 1080p for a balance of memory storage and resolution.

To enhance the accuracy and compensate for the errors from the common lens, the RSC method was applied. A specially designed ROC made of polylactide (PLA) was adhered on the specimen in the same plane of the surface (Figure 1). The ROC moved rigidly with the test specimen during loading and unloading. By compensating for the error with the displacement calculated on the ROC, we obtained the correct value of Poisson’s ratio in the cyclic test.

### 2.3. Transformation from Green Strain to Biot Strain and Hencky Strain under Uniaxial Tension

The output of Ncorr is the Green strain, a general description of strain tensor which can represent a rigid rotation of an unstrained body without producing any strain. However, the Biot strain and Hencky strain are more frequently used in practice. To transform the strain tensor, we start from the definition of Green strain as follows [29]:

(1) $$e_{xx}^{\left(G\right)}=\frac{1}{2}\left[2\frac{\partial u}{\partial x}+{\left(\frac{\partial u}{\partial x}\right)}^{2}+{\left(\frac{\partial v}{\partial x}\right)}^{2}\right]=\frac{1}{2}\left[2e_{xx}+{\left(e_{xx}\right)}^{2}+{\left(e_{xy}\right)}^{2}\right]$$

(2) $$e_{yy}^{\left(G\right)}=\frac{1}{2}\left[2\frac{\partial v}{\partial y}+{\left(\frac{\partial v}{\partial y}\right)}^{2}+{\left(\frac{\partial u}{\partial y}\right)}^{2}\right]=\frac{1}{2}\left[2e_{yy}+{\left(e_{yy}\right)}^{2}+{\left(e_{xy}\right)}^{2}\right]$$

(3) $$e_{xy}^{\left(G\right)}=\frac{1}{2}\left(\frac{\partial u}{\partial y}+\frac{\partial v}{\partial x}+\frac{\partial u\partial u}{\partial x\partial y}+\frac{\partial v\partial v}{\partial x\partial y}\right)=e_{xy}\left[1+\frac{1}{2}\left(e_{xx}+e_{yy}\right)\right]$$

where *∂u*/*∂x* ≡ *e_xx_* and *∂v*/*∂y* ≡ *e_yy_* are the infinitesimal strains in the *x* and *y* directions, respectively; *∂u*/*∂y = ∂v*/*∂x* ≡ *e_xy_* for isotropic materials.

Green strain may be regarded as the sum of small strain terms and quadratic terms. The small strain terms possess all the desirable properties of Biot strain behavior. The quadratic terms give the Green strain’s property of rotation independence. Recall that in the case of uniaxial tension, the axial displacement $u=\left(L_{F}-L_{0}\right)x/L_{0}=\;x\;\Delta L/L_{0}$, where *L_0_* and *L_F_* are respectively the initial and final length. Therefore, the infinitesimal strain is equal to the Biot strain as follows:

(4) $$e_{xx}\equiv \frac{\partial u}{\partial x}=\frac{\Delta L}{L_{0}}\equiv e_{xx}^{\left(B\right)}$$

Additionally, the shear stain *e_xy_* is zero in principle. Thus Equations (1) and (2) become

(5) $$e_{xx}^{\left(G\right)}=\frac{1}{2}\left[2e_{xx}^{\left(B\right)}+{\left(e_{xx}^{\left(B\right)}\right)}^{2}\right]$$

(6) $$e_{yy}^{\left(G\right)}=\frac{1}{2}\left[2e_{yy}^{\left(B\right)}+{\left(e_{yy}^{\left(B\right)}\right)}^{2}\right]$$

Biot strains can then be calculated using the measured Green strains. Note that when the strains are small, the quadratic terms are negligible and the Green strain is equal to the Biot strain.

For any given stretch ratio *a* = *L_F_/L*_0_ > 0, we define the nonlinear strain [30]

(7) $$e_{n}(a)=\{\begin{array}{ccc} \ln\left(a\right) & \mathrm{if} & n=0,\\ \frac{a^{n}-1}{n} & \mathrm{if} & n\neq 0, \end{array}$$

where *n* = 0, 1, and 2 represent Hencky strain *e*^(H)^ = *e*_0_ [31], Biot strain *e*^(B)^ = *e*_1_, and Green strain *e*^(G)^ = *e*_2_, respectively. For a uniaxial tension in the *x* direction, the Hencky strains (*n* = 0) are $e_{xx}^{\left(H\right)}=\ln(a)$ and $e_{yy}^{\left(H\right)}=\ln(\lambda )$, and the Biot strains (*n* = 1) are $e_{xx}^{\left(B\right)}=a-1$ and $e_{yy}^{\left(B\right)}=\lambda -1$, where *λ* is the stretch ratio in the transverse direction. *a* and *λ* can be determined from the known Biot strains, and are used to calculate the Hencky strains.

Hencky strain possesses a unique property that is useful for analyzing the volume change of a material undergoing large deformation. For compressible materials, $e_{xx}^{\left(H\right)}+e_{yy}^{\left(H\right)}+e_{zz}^{\left(H\right)}=e_{\mathrm{Vol}}^{\mathrm{True}}$, where the volume change $e_{\mathrm{Vol}}^{\mathrm{True}}=\int \left(1/V\right)dV=\ln\left(V_{F}/V_{0}\right)$ and *V*, *V*_0_, and *V_F_* denote the intermediate, initial, and final volume of the material, respectively. For incompressible materials, whose volume does not change during deformation, $e_{xx}^{\left(H\right)}+e_{yy}^{\left(H\right)}+e_{zz}^{\left(H\right)}=0$. Unlike Biot strains and Green strains, this relationship is valid even for large strains. In the present study, we use Hencky strain as the primary measure of strain for analyzing nonlinear Poisson’s ratios following Pritchard et al. [32].

### 2.4. Nonlinear Poisson’s Ratios

The nonlinear Poisson’s ratios are defined as follows [17]:

(8) $$\nu_{n}\left(a\right)=-\frac{e_{n}\left(\lambda \left(a\right)\right)}{e_{n}\left(a\right)}$$

where *e_n_* represents the various strain measures defined in Equation (7). Similarly, they are referred to as Hencky form with $\nu_{}^{\left(H\right)}\left(a\right)=\nu_{0}^{}\left(a\right)$, Biot form with $\nu_{}^{\left(B\right)}\left(a\right)=\nu_{1}^{}\left(a\right)$, and Green form with $\nu_{}^{\left(G\right)}\left(a\right)=\nu_{2}^{}\left(a\right)$. For infinitesimal deformation as *a* approaches 1, all these coincide with the Poisson’s ratio from the linear elastic theory. Note that for incompressible materials, only the Hencky form remains constant at 0.5 during deformation, and captures the characteristic of volume conservation for incompressible materials.

## 3. Results and Discussion

### 3.1. Transformation from the Green Strain to Biot Strain and Hencky Strain under Uniaxial Tension

**Figure 2:**
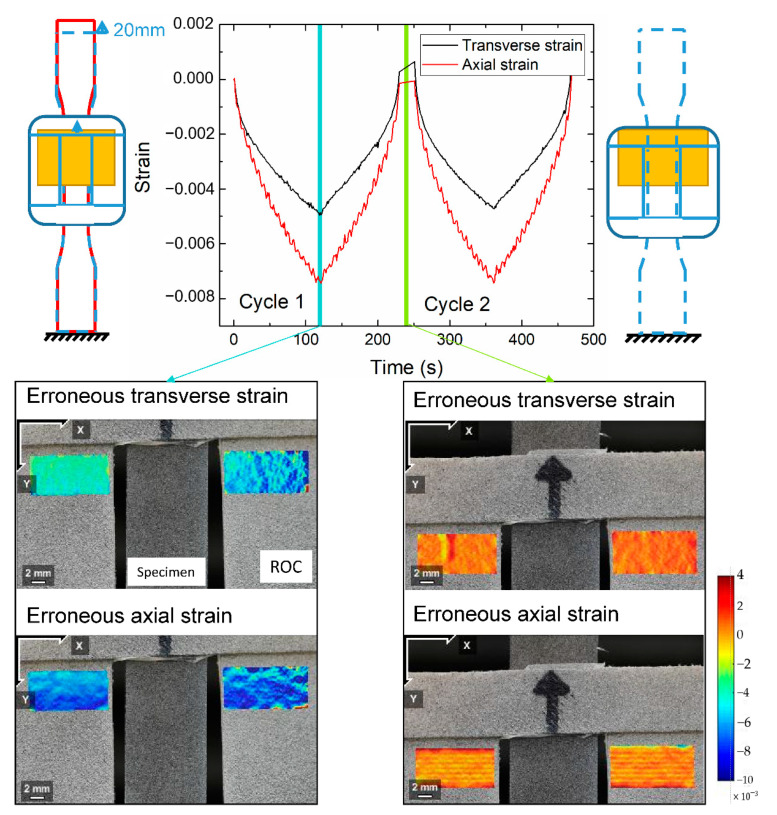
Erroneous Green strains measured on the ROC due to the out-of-plane motion over two loading–unloading cycles. The erroneous strains increase with the stretch, and return to zero when the specimen is unloaded. This phenomenon is evident from the DIC measurement on the undeformed ROC. The left and right panels were taken at stretch ratios of 1.186 and 1.0188, respectively.

**Figure 3:**
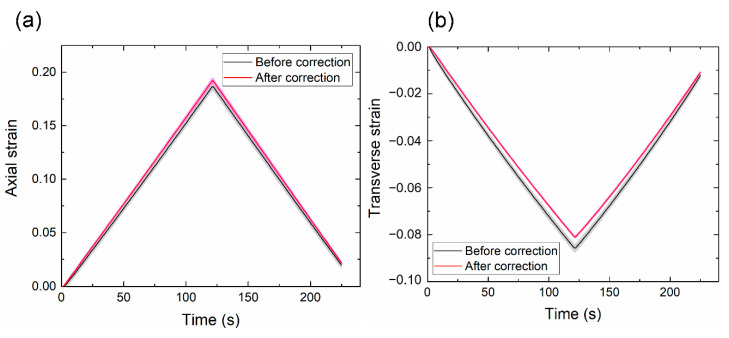
Means and standard deviations of the (**a**) axial strain and (**b**) transverse strain at a speed of 10 mm/min. The shaded areas represent one standard deviation of six samples. The axial and transverse strains were measured simultaneously on the same samples.

**Figure 4:**
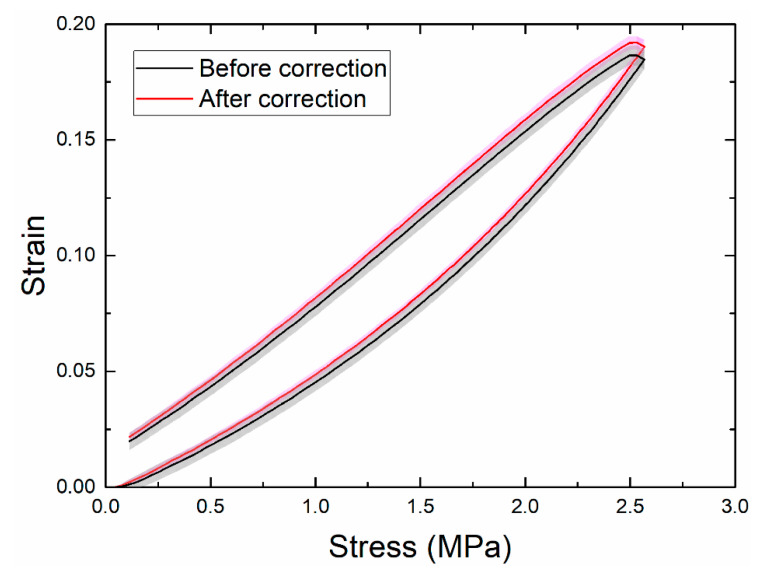
Means and standard deviations of the axial-strain curve at a speed of 10 mm/min. The shaded areas represent one standard deviation of six samples.

The image quality greatly affects the accuracy of DIC analysis. Unlike those captured with a bilateral telecentric lens, a photo captured by a normal lens is sensitive to the out-of-plane motion of the test specimen. When the specimen moves away from its initial position, the image of the specimen becomes smaller. As a result, DIC may produce an erroneous negative strain. When subjected to an axial tension in the *y* direction, the specimen contracts in the *x* and *z* directions if the material has a positive Poisson’s ratio. The out-of-plane motion is caused by the contraction in the *z* direction (away from the camera). Other inevitable measurement imperfections, including camera tilt and lens distortion, may also cause out-of-plane motion. For example, Figure 2 shows that at a stretch ratio of 118%, DIC gave non-zero strains on the undeformed ROC surface due to out-of-plane motion; these erroneous strains were of magnitudes of approximately −0.8% and −0.5% in the axial and transverse directions, respectively. These values may appear small compared to the stretch, but in the following section we show that they significantly affected the accuracy of the Poisson’s ratio calculation. Figure 3 shows that axial and transverse strains versus time before and after the correction for the first cycle. Without the correction, both strains were smaller than the actual values due to the out-of-plane motion. A comparison of the equilibrium stress-strain curves also showed a considerable discrepancy between the uncorrected and the corrected cases (Figure 4). Thus, compensation of such errors, particularly in large deformations, is necessary for accurate measurements using 2D-DIC [24,25].

### 3.2. Nonlinear Poisson’s Ratios Represented by Biot Strain and Hencky Strain

**Figure 5:**
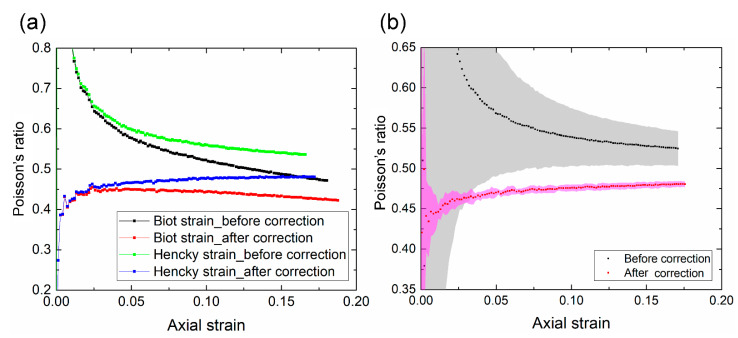
(**a**) Comparison of the Poisson’s ratios determined by Biot and Hencky strains before and after the correction for the first loading. (**b**) Means and standard deviations of the Poisson’s ratio (Hencky form). The results are based on six samples at a loading rate of 10 mm/min.

We conducted tension tests using six TPU specimens, and analyzed their strains and Poisson’s ratios during the loading process (Figure 5). A careful investigation highlighted the following observations: first, the uncorrected and corrected average Poisson’s ratios (both Biot and Hencky forms) exhibited distinct patterns (Figure 5a). The uncorrected values were well above 0.5 and even larger at the beginning of the loading where the strains were small. This is unreasonable for an isotropic linearly elastic material. After they were corrected with the RSC method, however, the Poisson’s ratios of both forms dropped below 0.5 and are expected to be more accurate in representing the actual material behavior. Second, the corrected Poisson’s ratio in the Biot form coincided with that in the Hencky form for small strains (<2.5%). They deviated from each other as the strain increased—the Biot form slightly decreased, whereas the Hencky form slightly increased. Third, the corrected Poisson’s ratio exhibited a much smaller standard deviation than the uncorrected one, demonstrating the repeatability of the RSC method (Figure 5b). High repeatability is critical when analyzing TPUs undergoing large deformations in a cyclic test.

### 3.3. Verification of the Quasi-Static Condition of the Cyclic Test

**Figure 6:**
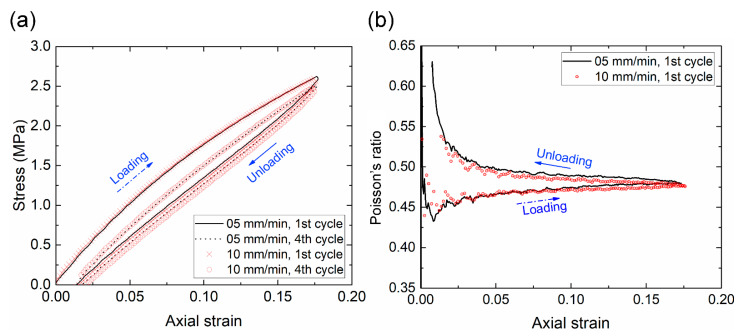
(**a**) The equilibrium stress-strain curves at the first and fourth cycles at loading rates of 5 mm/min and 10 mm/min. (**b**) Effect of the loading rate on the Poisson’s ratio (Hencky form) at the first cycle.

To ensure that the measurement was conducted in a quasi-static state, we compared the responses using two different rates: 5 mm/min and 10 mm/min. The former is recommended by the ASTM D638 standard test for the tensile properties of plastics. The stress-strain curves of both rates overlapped with each other for the first cycle and the fourth cycle (Figure 6a). The Poisson’s ratios (Hencky form) measured by DIC at the first cycle were also similar at both rates (Figure 6b). This verified that the measurement at the rate of 5 mm/min was indeed in a quasi-static state. Thus, we can neglect the influence of strain-rate dependence, which is common in viscoelastic materials but is beyond the scope of the present study.

### 3.4. Changes in Poisson’s Ratio during Cyclic Softening

**Figure 7:**
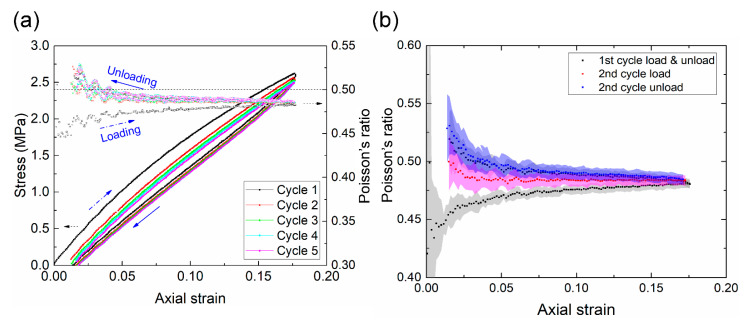
(**a**) The variations of the stress-strain curves and Poisson’s ratio in the cyclic test. (**b**) Means and standard deviations (shaded area) of the Poisson’s ratio (by Hencky strain) at the first and second cycles. The results are based on six samples at a loading rate of 10 mm/min.

A hysteresis loop was observed in the equilibrium stress-strain curve, indicating that part of the strain energy dissipated during the loading-unloading cycle, which is common in viscoelastic materials. The decrease of stress after the first cycle is known as cyclic softening, which has been investigated since first reported by Mullins in 1969 [13]. Figure 7a shows that the cyclic softening reached equilibrium after the fourth cycle, which is consistent with prior works [12]. Interestingly, we found that the Poisson’s ratio exhibited a hysteresis loop in the first cycle and remained largely constant at 0.48 afterward. This pattern appears to correlate with that of cyclic softening, and has not been reported before.

To investigate the changes of the Poisson’s ratio in more detail, we plotted the means and standard deviations of the Poisson’s ratios of six samples at the first two cycles (Figure 7b). We highlight three key observations. First, the standard deviation was generally small (e.g., ≈0.005 at a strain of 10%), indicating that the DIC and RSC method is robust and repeatable. However, it was very large at small strains (e.g., <1%) due to low signal-to-noise ratios. Thus, the data in the small strain regime are not reliable. Second, similar to the equilibrium stress-strain curve, a clear hysteresis loop of the Poisson’s ratio was present in the first cycle, as observed in Figure 7a. The Poisson’s ratio started at a relatively low value of 0.45, and gradually increased with the strain until reaching a relatively high value of 0.48 at the maximum strain (≈17.5%). It remained at a high value during the unloading process. Third, the degree of hysteresis decreased in the second cycle; the Poisson’s ratio during loading was also smaller than that during unloading.

As pointed out previously, the cyclic softening is correlated with the hysteresis loop of the Poisson’s ratio (Figure 7a). For simplicity, we assumed that the Poisson’s ratios were respectively 0.45 and 0.48 for the loading and unloading processes of the first cycle. The volume change of the specimen in the first loading (from 0 to a strain of 17.5%) can then be estimated by

(9) $$\ln\left(V_{F}/V_{0}\right)=e_{\mathrm{Vol}}^{\mathrm{True}}=e_{xx}^{\left(H\right)}+e_{yy}^{\left(H\right)}+e_{zz}^{\left(H\right)}=0.175-0.175\times 0.45\times 2=0.0175,$$

that is, *V_F_*/*V*_0_ ≈ 1.018. Similarly, the volume change in the first unloading was estimated to be approximately 0.993. Thus, the overall volume change in the first cycle was 1.018 × 0.993 = 1.011. That is, the phenomenon of cyclic softening (the Mullins effect) was accompanied by a slight increase in volume (≈1%). Our new experimental evidence may be helpful in understanding the physical source of this important phenomenon.

## 4. Conclusions

In this work, we demonstrated the application of 2D-DIC and RSC for measuring the nonlinear Poisson’s ratio of highly deformed TPU during a cyclic tensile test, and paid particular attention to the cyclic softening phenomenon. We found that RSC was essential to improve the accuracy and precision of 2D-DIC in this case. The uncorrected 2D-DIC produced significant errors in the Poisson’s ratio (+15%). With the RSC method, repeatable measurements of nonlinear Poisson’s ratio in large strains could be obtained by a low-cost 2D-DIC system without an expensive bilateral telecentric lens. In our experiments, the Poisson’s ratio of TPU exhibited a hysteresis loop, accompanied by cyclic softening, in the first cycle, and remained largely constant at 0.48 afterward. This change of the Poisson’s ratios resulted in a slight volume increase (≈1%) at a maximum strain of 17.5%. Our method has the merits of high resolution and high precision without the need of expensive instruments, and can be readily applied to other soft materials. However, it is less effective if the specimen experiences certain buckling deformation during compression. Our findings may be useful for those who use finite element method to analyze the performance of soft robots, and shed new light on understanding the physical origin of cyclic softening in rubber-like materials. The present study focuses on the quasi-static behavior of TPU. Since TPU is a viscoelastic material, it is expected that its Poisson’s ratio also varies with time and temperature [33,34,35]. How the strain rate and temperature affect the nonlinear Poisson’s ratio in the cyclic test is a topic for future work.

## Data Availability

The data presented in this study are available on request from the corresponding author.
